# Supplementary material for: A short linear motif, conserved from yeast to human, binds to members of the Spa2 family of cortical scaffold proteins
Source: J Cell Sci. 2026 Jan 13;139(1):jcs264236. doi: 10.1242/jcs.264236 (PMC12831199; doi:10.1242/jcs.264236)
Supplement: Supplementary information [file joces-139-264236-s1.pdf]

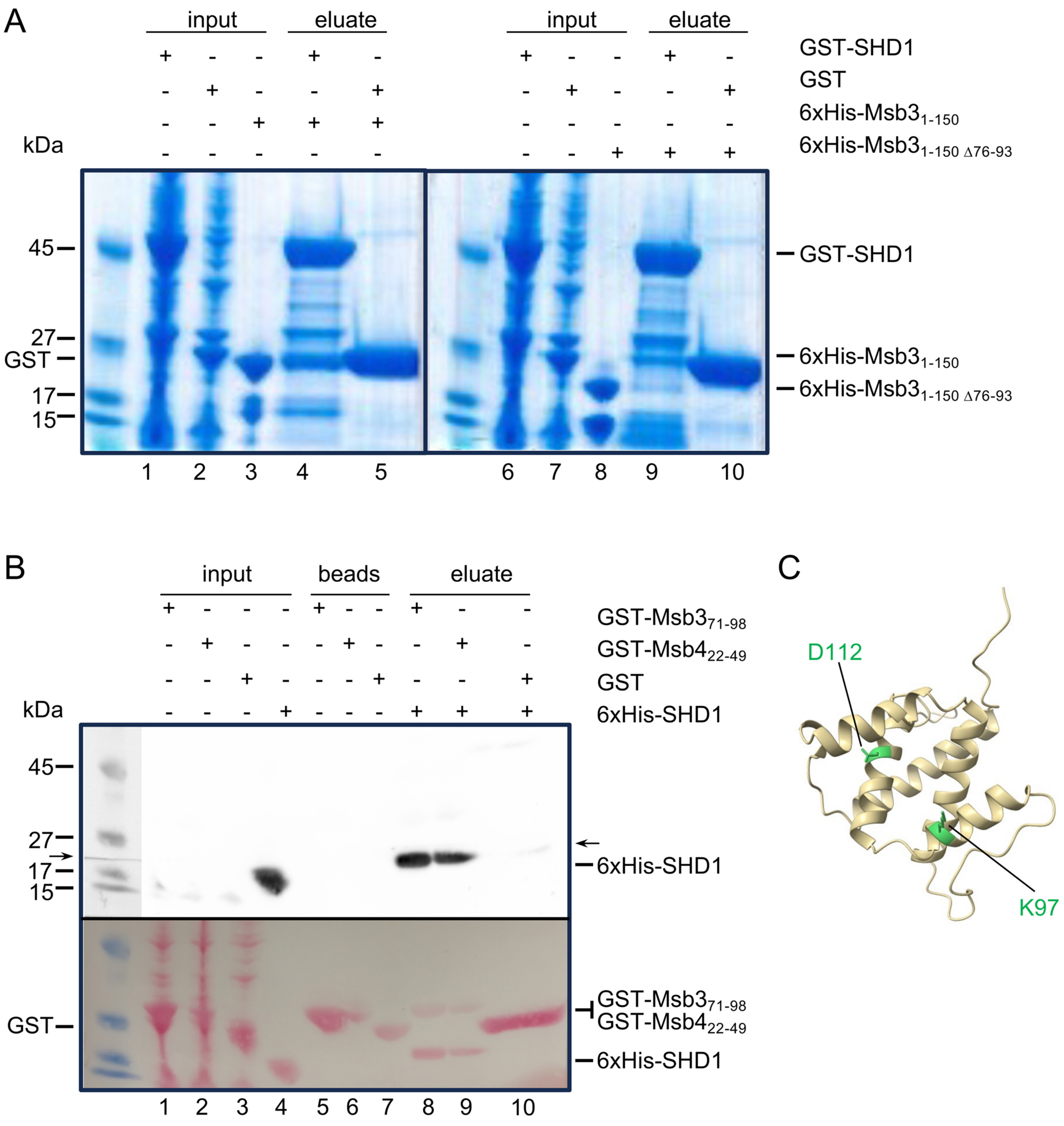

Fig. S1. (A) Inputs for Fig.1 G. Coomassie staining of SDS-PAGE of extracts of *E.coli* cells expressing GST-SHD1 (lanes 1, 6) or GST (lanes 5, 10), the enriched 6xHis-Msb3$_{1\text{-}150}$ (lane 3), or 6xHis-Msb3$_{1\text{-}150\Delta 76\text{-}93}$ (lanes 8) and the eluates of GST-SHD1- (lane 4, 9) or GST- (lanes 5, 10) coupled beads after being incubated with the enriched 6xHis-Msb3$_{1\text{-}150}$ (lanes 4, 5), or 6xHis-Msb3$_{1\text{-}150\Delta 76\text{-}93}$ (lanes 9, 10). (B) Full analysis of the GST pull-down experiment shown in Figure 1I. Lanes 1–4: input; lanes 5–7: SDS eluate after glutathione eluate; lanes 8–10: glutathione eluate. Lanes 8–10 are identical to lanes 1–3 in Figure 1I. Upper panel: anti-His immunoblot. Arrow indicates position of the cut. Lower panel: Ponceau S stain of the same membrane before antibody incubation. (C) AlphaFold structure prediction of SHD1 from yeast.

Journal of Cell Science • Supplementary information

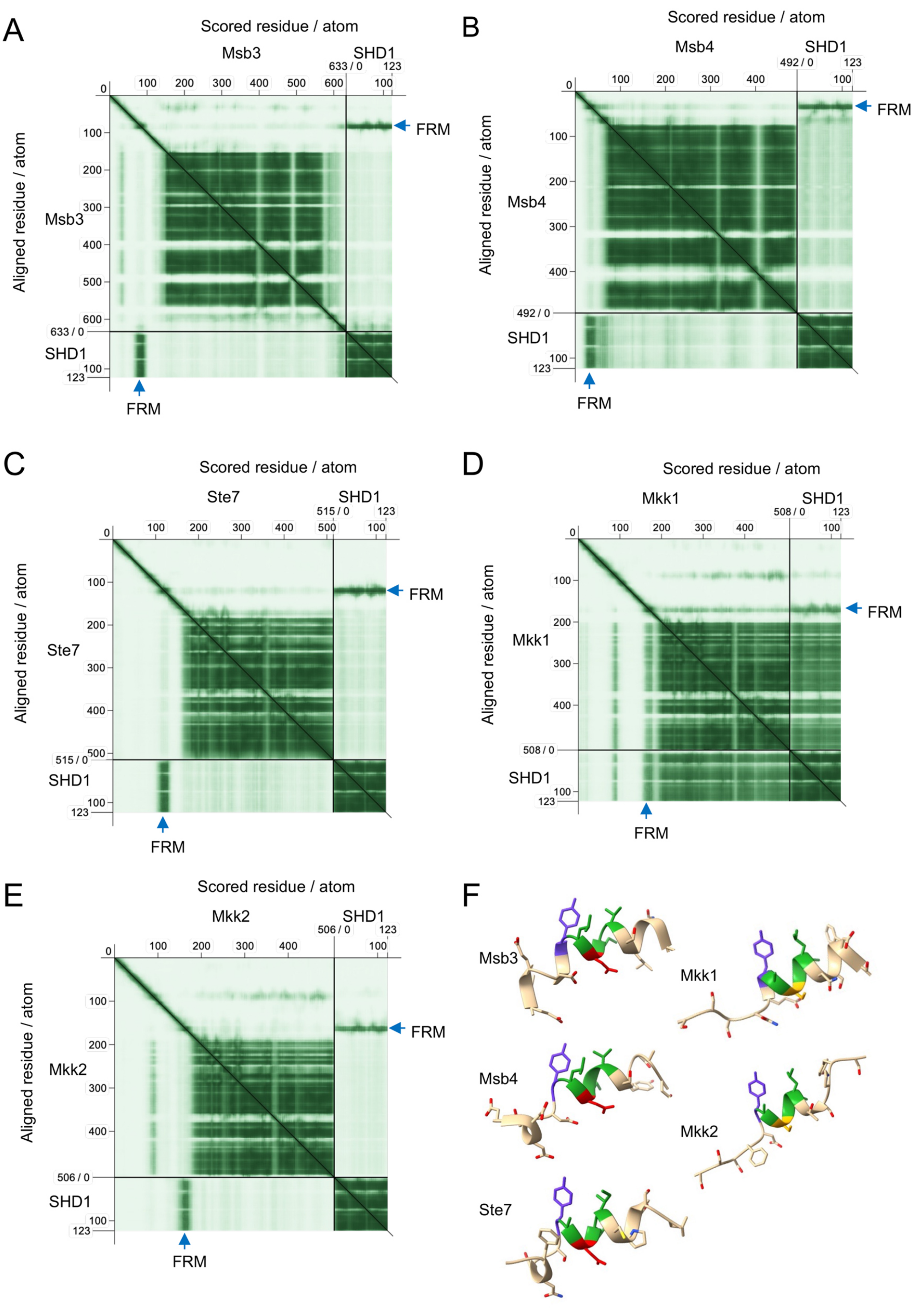

Fig. S2. (A-E) Predicted Aligned Error (PAE) matrices from the AlphaFold predictions of the complexes between the SHD1 of budding yeast and the full length Msb3 (A), Msb4 (B), Ste7 (C), Mkk1 (D) and Mkk2 (E). Blue arrows point to the sequences around the FRMs. Dark green indicates a high probability score of interaction with the SHD1. (G) Predicted structures of the FRM-containing sequences of the complexes of A-E.

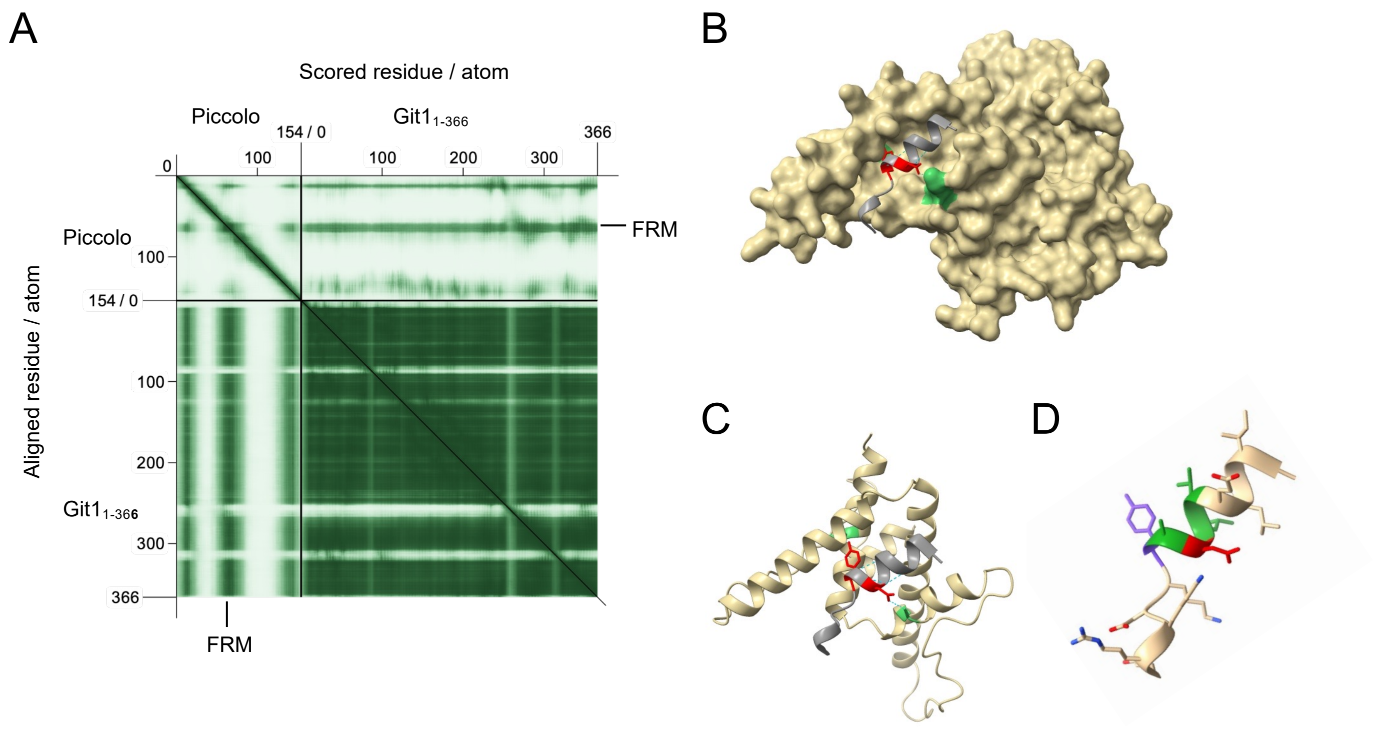

Fig. S3.

(A) Predicted Aligned Error (PAE) matrices from the AlphaFold predictions of the complexes between the GIT1$_{1\text{-}366}$ of rat and a fragment of Piccolo. Blue arrows points to the sequences around the FRMs that display a high score of interaction with the SHD1 of GIT1. (B) AlphaFold structure of the complex between GIT$_{1\text{-}366}$ and the FRM of Piccolo indicated in grey and highlighting the Y and D at the central positions. (C) Structure of the complex between the SHD1 of GIT1 and the FRM of Piccolo.

(D) Structure of the helix containing the FRM of Piccolo.

Journal of Cell Science • Supplementary information

Source File: Figure 1G

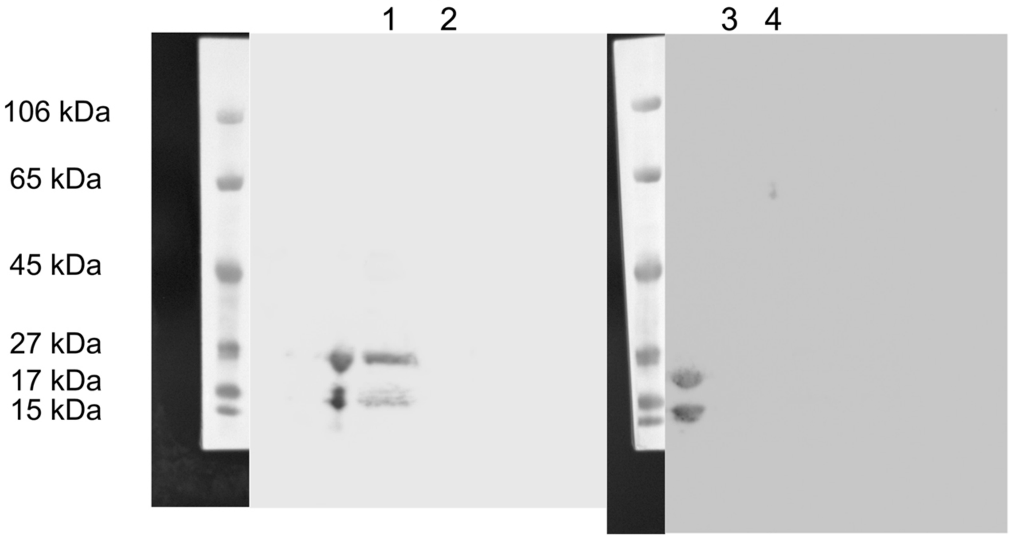

Source File: Figure S1B Figure 1I

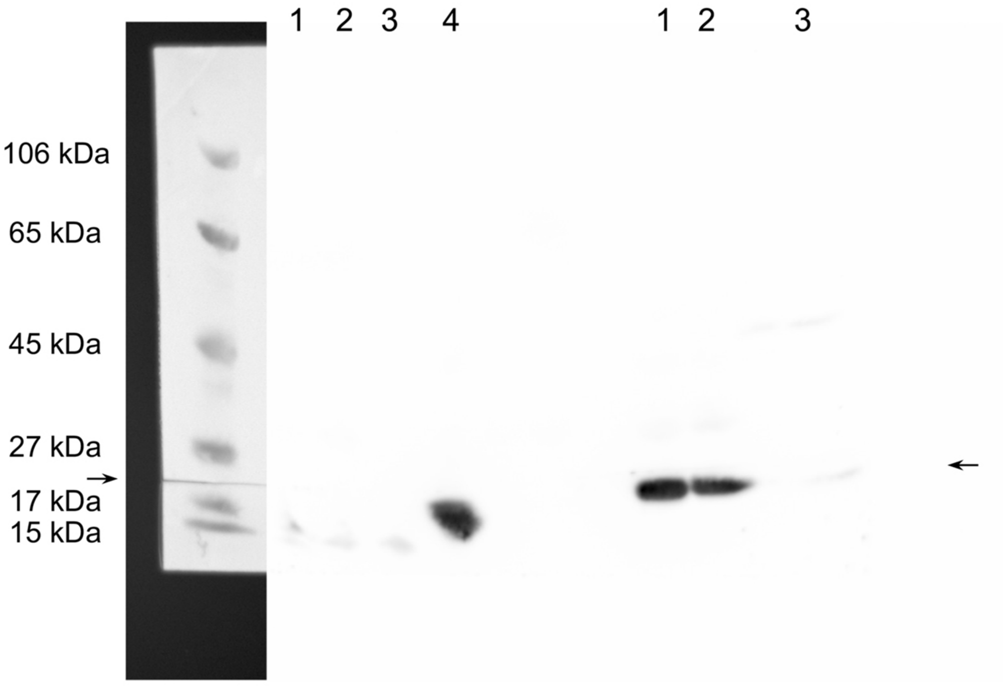

Source File: Figure 6B

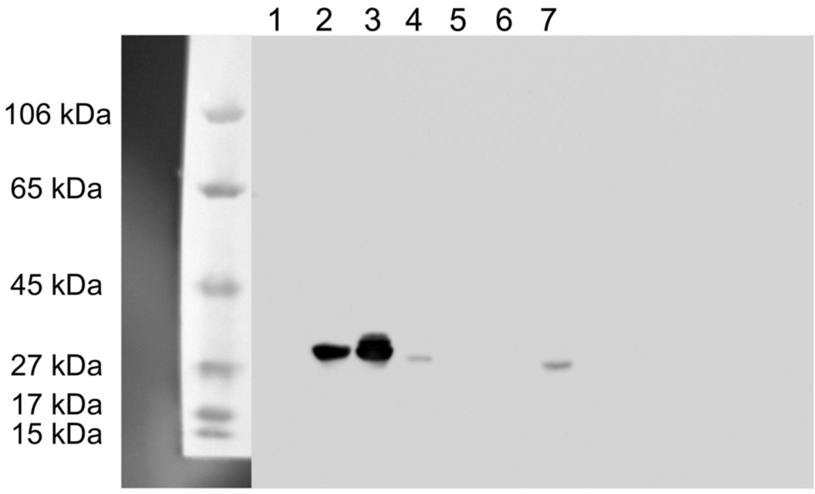

Fig. S4.

Table S1. List of *S. cerevisiae* strains used and created in this study.

Identifier	Strain	Genotype	Source
JD47	JD47	*MATa; his3-Δ200; leu2-3, 112; lys2-801; trp1Δ63; ura3-52*	(Dohmen et al., 1995)
JD53	JD53	*MATα; his3-Δ200; leu2-3, 112; lys2-801; trp1Δ63; ura3-52*	(Dohmen et al., 1995)
YAD248	spa2Δ	*SPA2::hphNT1, JD47*	Johnsson Lab
YAD2703	msb3Δ; msb4Δ	*MSB3::hphNT1, msb4::kanMX, JD47*	Johnsson Lab
YAD2586	msb3Δ	*MSB3::natNT2, JD47*	Johnsson Lab
YAD2588	msb4Δ	*MSB4::kanMX4, JD47*	Johnsson Lab
YAD3097	spa2$_{Δ2\text{-}150}$	*spa2*$_{Δ2\text{-}150}$*, JD47*	Johnsson Lab
YLB53	msb3$_{Δ76\text{-}93}$	*msb3*$_{Δ76\text{-}93}$*, JD47*	This study
YAD4313	msb4$_{Δ27\text{-}44}$	*msb4*$_{Δ27\text{-}44}$*, JD47*	This study
YLB55	msb3Δ; msb4$_{Δ27\text{-}44}$	*MSB3::natNT2, msb4*$_{Δ27\text{-}44}$*, JD47*	This study
YLB61	msb4Δ; msb3$_{Δ76\text{-}93}$	*MSB4::kanMX4, msb3*$_{Δ76\text{-}93}$*, JD47*	This study
YAD4580	ste7Δ	*STE7::natNT2, JD47*	Johnsson Lab
YAD4570	ste7$_{Δ119\text{-}123}$	*ste7*$_{Δ119\text{-}123}$*, JD47*	Johnsson Lab
NBY167	N$_{ub}$-Msb3	P_{MSB3}*::kanMX4* P_{CUP1} N_{Ub}*-HA, JD53*	Johnsson Lab
NBY176	N$_{ub}$-Msb4	P_{MSB4}*::kanMX4* P_{CUP1} N_{Ub}*-HA, JD53*	Johnsson Lab
NBY22	N$_{ub}$-Bud2	P_{BUD2}*::kanMX4* P_{CUP1} N_{Ub}*-HA, JD53*	Johnsson Lab
NBY31	N$_{ub}$-Bud3	P_{BUD3}*::kanMX4* P_{CUP1} N_{Ub}*-HA, JD53*	Johnsson Lab
NJY194	N$_{ub}$-Ste7	P_{STE7}*::kanMX4* P_{CUP1} N_{Ub}*-HA, JD53*	Johnsson Lab
NBY270	N$_{ub}$-YEL023C	$P_{YEL023C}$*::kanMX4* P_{CUP1} N_{Ub}*-HA, JD53*	Johnsson Lab
YAD4299	N$_{ub}$-Msb3$_{Δ84\text{-}88}$	P_{MSB3}*::kanMX4* P_{CUP1} N_{Ub}*-HA, MSB3::msb3*$_{Δ84\text{-}88}$*, JD53*	This study
YAD4300	N$_{ub}$-Msb4$_{Δ35\text{-}39}$	P_{MSB4}*::kanMX4* P_{CUP1} N_{Ub}*-HA, MSB4::msb4*$_{Δ35\text{-}39}$*, JD53*	This study
YAD4303	N$_{ub}$-Ste7$_{Δ119\text{-}123}$	P_{STE7}*::kanMX4* P_{CUP1} N_{Ub}*-HA, STE7::ste7*$_{Δ119\text{-}123}$*, JD53*	This study
NBY339	N$_{ub}$-Guk1	P_{GUK1}*::kanMX4* P_{CUP1} N_{Ub}*-HA, JD53*	Johnsson Lab

Journal of Cell Science • Supplementary information

MZY16	N$_{ub}$ empty	P_{CUP1} N_{Ub}-HA, JD53	Johnsson Lab
NBY156	N$_{ub}$-Spa2	P_{SPA2}::kanMX4 P_{CUP1} N_{Ub}-HA, JD53	Johnsson Lab
NBY673	N$_{ub}$-Spa2$_{\Delta 2\text{-}188}$	$P_{SPA2\Delta 2\text{-}188}$::kanMX4 P_{CUP1} N_{Ub}-HA, JD53	Johnsson Lab
NBY674	N$_{ub}$-Spa2$_{\Delta 2\text{-}150}$	$P_{SPA2\Delta 2\text{-}150}$::kanMX4 P_{CUP1} N_{Ub}-HA, JD53	Johnsson Lab
NBY665	N$_{ub}$-Spa2$_{\Delta 2\text{-}188}$	$P_{SPA2\Delta 2\text{-}188}$::kanMX4 P_{CUP1} N_{Ub}-HA, JD53	Johnsson Lab
NBY787	N$_{ub}$-Msb3$_{L82Y}$	$P_{MSB3L82Y}$::kanMX4 P_{CUP1} N_{Ub}-HA, JD53	This study
NBY785	N$_{ub}$-Msb3$_{V84A}$	$P_{MSB3V84A}$::kanMX4 P_{CUP1} N_{Ub}-HA, JD53	This study
NBY774	N$_{ub}$-Msb3$_{I85A}$	$P_{MSB3I85A}$::kanMX4 P_{CUP1} N_{Ub}-HA, JD53	This study
NBY776	N$_{ub}$-Msb3$_{D86A}$	$P_{MSB3D86A}$::kanMX4 P_{CUP1} N_{Ub}-HA, JD53	This study
NBY782	N$_{ub}$-Msb3$_{L87A}$	$P_{MSB3L87A}$::kanMX4 P_{CUP1} N_{Ub}-HA, JD53	This study
NBY778	N$_{ub}$-Msb3$_{Y88A}$	$P_{MSB3Y88A}$::kanMX4 P_{CUP1} N_{Ub}-HA, JD53	This study
NBY780	N$_{ub}$ -Msb3$_{Y88F}$	$P_{MSB3Y88F}$::kanMX4 P_{CUP1} N_{Ub}-HA, JD53	This study
NBY703	N$_{ub}$-Msb4$_{D37S}$	$P_{MSB4D37S}$::kanMX4 P_{CUP1} N_{Ub}-HA, JD53	This study
NBY704	N$_{ub}$-Msb4$_{D37K}$	$P_{MSB4D37K}$::kanMX4 P_{CUP1} N_{Ub}-HA, JD53	This study
NBY715	N$_{ub}$-Msb4$_{Y39A}$	$P_{MSB4Y39A}$::kanMX4 P_{CUP1} N_{Ub}-HA, JD53	This study
NBY107	N$_{ub}$-Epo1	P_{EPO1}::kanMX4 P_{CUP1} N_{Ub}-HA, JD53	Johnsson Lab
NBY595	N$_{ub}$-Aip5	P_{AIP5}::kanMX4 P_{CUP1} N_{Ub}-HA, JD53	Johnsson Lab
NBY450	N$_{ub}$-Pea2	P_{PEA2}::kanMX4 P_{CUP1} N_{Ub}-HA, JD53	Johnsson Lab
NBY211	N$_{ub}$-Mkk1	P_{MKK1}::kanMX4 P_{CUP1} N_{Ub}-HA, JD53	Johnsson Lab
NBY734	N$_{ub}$-Mkk1$_{L171V}$	$P_{MKK1L171V}$::kanMX4 P_{CUP1} N_{Ub}-HA, JD53	This study
NBY705	N$_{ub}$-Mkk1 S173D	$P_{MKK1173D}$::kanMX4 P_{CUP1} N_{Ub}-HA, JD53	This study
NBY139	N$_{ub}$-Ste11	P_{STE11}::kanMX4 P_{CUP1} N_{Ub}-HA, JD53	This study
NBY767	N$_{ub}$-Ste11$_{\Delta 1\text{-}105}$	$P_{STE11\Delta 1\text{-}105}$::kanMX4 P_{CUP1} N_{Ub}-HA, JD53	This study
NBY754	N$_{ub}$-Ste11$_{\Delta 1\text{-}410}$	$P_{STE11\Delta 1\text{-}410}$::kanMX4 P_{CUP1} N_{Ub}-HA, JD53	This study
N0BY73	N$_{ub}$-Ste7Δ1-164	$P_{STE7\Delta 1\text{-}164}$::kanMX4 P_{CUP1} N_{Ub}-HA, JD53	This study
STY165	Msb3$_{\Delta 76\text{-}93}$-CRU 303	MSB3$_{\Delta 76\text{-}93}$::msb3-C_{ub}-R-URA3 HIS3, JD47	Johnsson Lab
YLB58	Msb3$_{\Delta 76\text{-}93}$-CRU 303	msb3 $_{\Delta 76\text{-}93}$::msb3-C_{ub}-R-URA3 HIS3, JD47	This study

Journal of Cell Science • Supplementary information

YAD4440	pMet17 Msb4-CRU 303	*P_{MSB4}::P_{MET17}, MSB4::msb4-C_{ub}-R-URA3 HIS3, JD47*	Johnsson Lab
YAD4441	pMet17 Msb4$_{\Delta 27\text{-}44}$-CRU 303	*P_{MSB4}::P_{MET17}, msb4$_{\Delta 27\text{-}44}$::msb4-C_{ub}-R-URA3 HIS3, JD47*	This study
UNY7	Ste5-CRU303	*ste5::ste5-C_{ub}-R-URA3 HIS3, JD47*	Johnsson Lab
STY1659	Ste7$_{105\text{-}165}$-Spa2	*P_{SPA2}*::ste7*(105-165)-spa2::spa2-C_{ub}-R-URA3 HIS3, JD47*	Johnsson Lab
STY1657	Msb4$_{22\text{-}81}$-Spa2	*P_{SPA2}::msb4(22-81)-spa2::spa2-C_{ub}-R-URA3 HIS3, JD47*	Johnsson Lab
STY1658	Msb4$_{22\text{-}81\Delta 35\text{-}39}$ -Spa2	*P_{SPA2}::msb4(22-81)$_{\Delta 35\text{-}39}$-spa2::spa2-C_{ub}-R-URA3 HIS3, JD47*	Johnsson Lab
YAD2998	Msb3-GFP304	*MSB3:MSB3-GFP TRP1*	Johnsson Lab
YAD2999	Msb4-GFP304	*MSB3:MSB4-GFP TRP1*	Johnsson Lab
YAD4318	Msb3$_{\Delta 76\text{-}93}$-GFP 304	*MSB3::msb3$_{\Delta 76\text{-}93}$-GFP TRP1*	This study
YLB83	Msb4$_{\Delta 27\text{-}44}$-GFP 304	*MSB4::msb4$_{\Delta 27\text{-}44}$-GFP TRP1*	This study
YLB86	Msb3-GFP 304; Spa2$_{\Delta 2\text{-}150}$	*MSB3::msb3-GFP TRP1, SPA2::spa2$_{\Delta 2\text{-}150}$, JD47*	This study
YLB87	Msb4-GFP 304; Spa2$_{\Delta 2\text{-}150}$	*MSB4::msb4-GFP TRP1, SPA2::spa2$_{\Delta 2\text{-}150}$, JD47*	This study
YAD4500	Ste7-GFP 304	*STE7::ste7-GFP TRP1, JD47*	Johnsson Lab
YAD4501	Ste7$_{\Delta 119\text{-}123}$-GFP 304	*STE7::* ste7*$_{\Delta 119\text{-}123}$-GFP TRP1, JD47*	Johnsson Lab

Journal of Cell Science • Supplementary information

Table S2. List of plasmids used and created in this study.

Identifier	Plasmid	Genotype	Source
BS4075	PMET17-Spa2(1-124)-CRU pRS313	*PMET17-spa2(1-124)- Cub-R-URA3, AmpR, HIS3, CEN*	Johnsson Lab
BA2009	PMET17-Spa2 L48,51A (1-124) - CRU pRS313	*PMET17-spa2 L48,51A (1-124)- Cub-R-URA3, AmpR, HIS3, CEN*	Johnsson Lab
BL1001	PMET17-Spa2 L101A (1-124)-CRU pRS313	*PMET17-spa2 L101A (1-124)- Cub-R-URA3, AmpR, HIS3, CEN*	This study
BL1002	PMET17-Spa2 L109A (1-124)-CRU pRS313	*PMET17-spa2 L109A (1-124)- Cub-R-URA3, AmpR, HIS3, CEN*	This study
BL1003	PMET17-Spa2 L101,109A (1-124)- CRU pRS313	*PMET17-spa2 L101,109A (1-124)- Cub-R-URA3, AmpR, HIS3, CEN*	This study
BL1004	PMET17-Spa2 K97D (1-124)-CRU pRS313	*PMET17-spa2 K97D (1-124)- Cub-R-URA3, AmpR, HIS3, CEN*	This study
BA2075	Msb4(22-81)Δ35-39-Spa(1-124)-CRU	P_{MET17}*MSB4(22-81)Δ35-39-SPA2(1-124)-CRU313*	This study
BA2080	Msb4(22-81)Y39A-Spa2(1-124)-CRU	P_{MET17}*MSB4(22-81)*Y39A-*SPA2(1-124)-CRU313*	This study
BA2072	Mkk1(105-164)-Spa2(1-124)-CRU	P_{MET17}*MKK1(105-164)-SPA2(1-124)-CRU313*	This study
BA2045	Ste7(105-165)-Spa2(1-124)-CRU	P_{MET17}*STE7(105-165)-SPA2(1-124)-CRU313*	This study
BA2044	Msb3(71-130)-Spa2(1-124)-CRU	P_{MET17}*MSB3(71-130)-SPA2(1-124)-CRU313*	This study
BA2075	Msb4(22-81)-Spa2(1-124)-CRU	P_{MET17}*MSB4(22-81)-SPA2(1-124)-CRU313*	This study
BA884	N_{ub}-$Spa2_{1\text{-}150}$	P_{CUP}*NubHA-Spa2(1-150) kanMX CEN*	Johnsson Lab
BA2006	N_{ub}-$Msb3_{71\text{-}100}$	P_{Cup}*NubHAkanMX* $Msb3_{71\text{-}100}$ *CEN*	Johnsson Lab
BA2007	N_{ub}-$Msb4_{21\text{-}50}$	P_{Cup}*NubHAkanMX* $Msb4_{21\text{-}50}$ *CEN*	Johnsson Lab
BA2008	N_{ub}-$Ste7_{105\text{-}135}$	P_{Cup}*NuiHAkanMX* $Ste7_{105\text{-}135}$ *CEN*	Johnsson Lab

Journal of Cell Science • Supplementary information

Table S3. Raw fluorescence intensities of GFP fusions for calculating the fluorescence ratios in Fig. 4D.

**Msb3**			**Msb3$_{\Delta 76\text{-}93}$**		
Shmoo tip	Cytoplasm	background	Shmoo tip	Cytoplasm	Background
964,583	643,375	512,833	829,5	882,417	587,083
801,583	614,292	525,167	758,182	725,909	578,909
778,875	643,25	525,625	746,727	758	576,273
878,458	668,333	524,833	752,818	742,818	595,727
769,5	612,625	529,375	732,818	711,909	567,636
973,875	689,583	528	727,545	807	564,455
838,917	625,917	525,75	732,636	726	586,455
1035,167	694,375	531,292	755,545	762	541,727
966,708	676,083	531,792	813,727	820,091	576,818
858,708	658,833	544,333	831,545	801,727	545,273

**Msb4**			**Msb4$_{\Delta 27\text{-}44}$**		
Shmoo tip	Cytoplasm	background	Shmoo tip	Cytoplasm	background
1363,364	1094,364	788,818	1294,636	1343	835,273
1162,545	966,455	775	1317,273	1264	824,818
1442	1051,545	769,455	1112	1096,727	866,273
1279,818	1089	751,909	1212,909	1306,273	776,818
1435,364	1096,091	727	1212,818	1291,636	801,636
1454,273	1088,182	773,182	1257	1258,909	847,273
1343,125	991,625	801,75	1268,455	1251,727	826,818
1159,438	858,455	751,545	1246,091	1397,273	834,091
1642	1226,364	902,818	1209,75	1203,182	843,545
1667,455	1453,818	1069,909	1209,545	1104	856,75

**Ste7**			**Ste7$_{\Delta 112\text{-}133}$**		
Shmoo tip	Cytoplasm	background	Shmoo tip	Cytoplasm	background
958,792	706,042	533,167	763,333	640,417	541,708
885,75	661,375	542	661,625	683,417	530,583
840,083	696,917	542,581	646,708	635,042	543,208
909	666,25	540,292	702,167	699,667	538,5
785,125	649,083	553,708	748,625	663,333	524,458
721,167	606,792	530,625	685,667	664,583	529,458
981,208	672,833	541,458	658,083	668,5	531,042
926,333	627,292	545,875	687,75	638,792	537,333
908,167	651,583	544,708	696,708	657,25	518,958
862,125	671,875	529,833	640,125	666,083	523

Journal of Cell Science • Supplementary information

Table S4. List of potential new FRM-containing binding partners of Spa2

UniProt Accession	Yeast Gene	Matching Step
P04710	ADT1	Step3
P06784	STE7	Step6
P07264	LEU1	Step4
P07866	LTE1	Step4
P09032	GCD1	Step2
P0C5P0	YJL197C	Step4
P0CX94	YER190C	Step2
P0CX95	YEL077W-A	Step2
P0CX96	YGR296C-B	Step2
P0CX97	YPL283W-B	Step2
P0CX98	YPR204C-A	Step2
P0CX99	YFL068W	Step2
P0CY00	YLL066W-A	Step2
P0CY01	YLL067W-A	Step2
P13433	RPO41	Step3
P14772	BPT1	Step4
P14908	MTF1	Step2
P15274	AMD1	Step4
P18239	ADT2	Step3
P18412	*TEC1*	Step4

P19097	*FAS2*	Step4
P19263	*MED14*	Step4
P19658	*EXO70*	Step4
P20967	*ODO1*	Step3
P21595	*CP56*	Step3
P23255	*TAF2*	Step4
P23642	*VAN1*	Step4
P25046	*MED19*	Step4
P25351	*YCR3*	Step4
P25389	*KCC4*	Step4
P25569	*GID7*	Step2
P26725	*YUR1*	Step2
P27654	*TIP1*	Step4
P28273	*OXP1*	Step4
P32461	*DPH2*	Step4
P32490	*MKK1*	Step6
P32491	*MKK2*	Step5
P32496	*RPN12*	Step4
P32522	*PET309*	Step3
P32523	*PRP19*	Step2
P32862	*RGT1*	Step4
P32906	*MNS1*	Step4

Journal of Cell Science • Supplementary information

P32907	*ATO2*	Step4
P35189	*TAF14*	Step2
P36051	*MCD4*	Step4
P36083	*YKH5*	Step4
P38009	*ADE17*	Step2
P38041	*BOB1*	Step4
P38073	*EDS1*	Step4
P38207	*APN2*	Step2
P38249	*RPG1*	Step5
P38696	*ARP1*	Step4
P39079	*CCT6*	Step4
P39931	*SSP120*	Step4
P39985	*DPO5*	Step4
P39986	*SPF1*	Step4
P40004	*YEA4*	Step4
P40350	*ALG5*	Step4
P40509	*SEC28*	Step4
P40529	*AGE2*	Step5
P40552	*TIR3*	Step4
P40857	*PHS1*	Step4
P40986	*CDC1*	Step2
P43571	*BST1*	Step4

P43583	*BLM10*	Step4
P46682	*AP3B*	Step4
P46954	*SIP4*	Step4
P46956	*PHO86*	Step2
P47069	*MPS3*	Step2
P47101	*RBH2*	Step4
P47167	*MCM22*	Step4
P48566	*MSB3*	Step6
P49704	*PRP31*	Step4
P50273	*ATP22*	Step3
P53108	*YIP5*	Step4
P53170	*PDK2*	Step3
P53239	*COX18*	Step3
P53829	*CAF40*	Step4
P53901	*INN1*	Step2
P53909	*AAH1*	Step4
P53952	*YNL050C*	Step4
P54113	*ADE16*	Step2
P60010	*ACT1*	Step4
Q00764	*TPS1*	Step4
Q02457	*TBF1*	Step4
Q02732	*CFT19*	Step4

Q03210	*NGL3*	Step2
Q03233	*ADD37*	Step4
Q03305	*RMT2*	Step4
Q03920	*MTQ2*	Step2
Q04002	*SCC2*	Step4
Q04178	*HPT1*	Step2
Q04301	*VBA1*	Step4
Q04322	*GYL1*	Step4
Q04372	*TAP42*	Step4
Q04511	*UFO1*	Step4
Q04585	*YDR109C*	Step4
Q04602	*VBA4*	Step4
Q05791	*CDC123*	Step2
Q06058	*SEI1*	Step2
Q06142	*IMB1*	Step4
Q06598	*ARR3*	Step4
Q06632	*CFT1*	Step2
Q06704	*IMH1*	Step4
Q07376	*MCH1*	Step4
Q07527	*TRM3*	Step4
Q07830	*GPI13*	Step4
Q08179	*MDM38*	Step4

Journal of Cell Science • Supplementary information

Q08236	*AVO1*	Step4
Q08729	*DSE3*	Step6
Q12029	*FSF1*	Step4
Q12038	*SRO7*	Step2
Q12109	*SYWC*	Step4
Q12167	*RRG1*	Step3
Q12200	*NPC1*	Step4
Q12205	*MNL2*	Step4
Q12297	*TAF3*	Step4
Q12317	*MSB4*	Step6
Q12321	*MED1*	Step4
Q12511	*PDP1*	Step2
Q3E755	*YBR200W-A*	Step5
Q6Q560	*ISD11*	Step4
Q99207	*NOP14*	Step4
Q99222	*AFI1*	Step4
